# Ulcerative intestinal tuberculosis case as a complication of treatment by infliximab for intestinal Behçet's disease: A case report

**DOI:** 10.1097/MD.0000000000017652

**Published:** 2019-10-25

**Authors:** Yan Shen, Hai-fen Ma, Yan-li Yang, Jian-long Guan

**Affiliations:** a Rheumatology and Immunology Department; b Medical Imaging Department, Huadong Hospital, Fudan University, Shanghai 200040, PR China.

**Keywords:** Behçet's disease, infliximab, intestinal tuberculosis, intestinal ulcer

## Abstract

**Rationale:**

Intestinal Behçet's disease (BD) is characterized by intestinal ulcerations and gastrointestinal symptoms. Ulcerative intestinal tuberculosis (TB) is usually with dyspepsia, abdominal pain, vomiting, and weight loss. The 2 diseases exhibit similar clinical manifestations, but the most critical aspects of their clinical courses and required treatments are not at all similar.

**Patient concerns:**

We present a case in which a patient with intestinal Behçet's disease developed a de novo ulcerative intestinal TB infection after the start of anti-tumor necrosis factor-α treatment. This was despite histopathologic examination without caseous necrosis granuloma and negative for acid-fast staining and latent TB screen.

**Diagnoses:**

Intestinal Behçet's disease and intestinal TB.

**Interventions:**

The patient was treated with quadruple antituberculous chemotherapy, comprising rifapentine, isoniazid, ethambutol, and pyrazinamide.

**Outcomes:**

At follow-up about 3 months, the therapy of oral antituberculous drugs and thalidomide was continued and the patient's condition had stabilized.

**Lessons:**

This case illustrates the importance of closely monitoring patients who are on infliximab for possible onset of TB, even without abdominal symptoms, and with negative screening results for latent TB.

## 1 Introduction

Intestinal Behçet's disease (BD) is characterized by intestinal ulcerations and gastrointestinal symptoms. The prevalence of intestinal BD has been reported to be 3% to 60%, although it varies in different populations.^[1–4]^ Intestinal BD can sometimes cause life-threatening comorbidities such as intestinal perforation and massive bleeding.^[5]^ The etiology of BD is thought to be related to environmental factors. Microbial infection, such as mycobacterium tuberculosis (MTB), is considered to be an environmental trigger of BD.^[6]^ Ulcerative intestinal tuberculosis is usually secondary to pulmonary tuberculosis and symptoms include fever, dyspepsia, abdominal pain, vomiting, and weight loss. The 2 diseases exhibit similar clinical manifestations, but the critical aspects of their clinical courses and treatments are very different. We present here a case of a patient with intestinal BD who developed ulcerative intestinal TB secondary to infliximab treatment.

## 2 Case report

**Figure 1:**
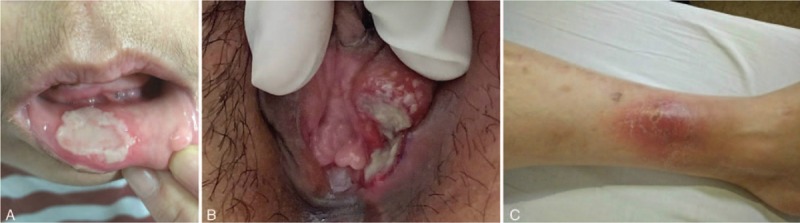
(A) oral ulcer; (B) genital ulcer; and (C) the lower limb of erythema nodosum.

A 44-year-old female presented to our hospital complaining of fever, oral ulcers, genital ulcers, and multiple erythema nodosum on limbs that had begun 2-weeks previously. She had oral aphthous ulceration in the past 1 year. It attacked 3 to 4 times a year, and last 1 to 2 weeks every time. She had not experienced other similar symptoms before. She had no symptoms of cough, weight loss, or night sweating and had no recurrent ophthalmia or vision loss. She had been previously well, without abdominal pain, distension, or vomiting. She did not have a history of tuberculosis or close contact with TB patients. There was no history of unusual travel or contaminated diet, contact with infected individuals, or antibiotic use. On physical examination, one large painful oral ulcerations (10 mm × 10 mm) and two painful genital ulcer (Fig. 1A and B). Erythema nodosum skin lesions were noted on her arms and legs, distributed on the extensor and flexor surfaces (Fig. 1C). Pathology test results were negative. Abdominal physical examination was normal.

**Figure 2:**
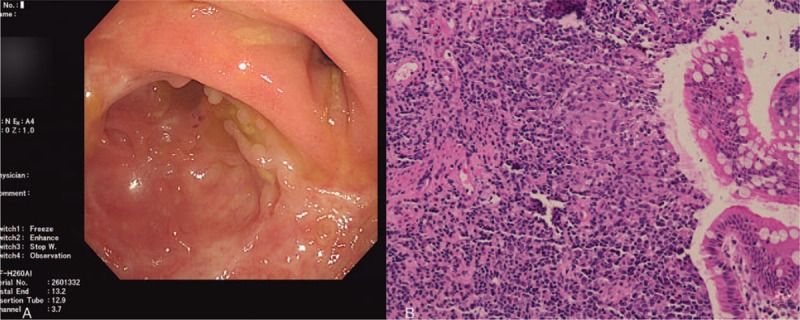
Colonoscopy finding and histopathologic examination before anti-tumor necrosis factor-α treatment. A, colonoscopic image of the patient showed dispersed irregular ulcers in cecum, ileocecus and ascending colon. B, histopathologic examination from the ascending colon ulcer shows mucosal medium lymphocyte, plasma cell, neutrophil infiltration, and erosion exudate.

Blood test results included the following: hemoglobin of 82.0 g/dl, indicative of microcytic hypochromic anemia; elevated erythrocyte sedimentation rate of 40 mm/h (normal range: <20 mm/h); and elevated C-reactive protein 22.3 mg/L (normal range: <10 mg/L). Tests for antinuclear antibodies, anti-double stranded DNA, anti-extractable nuclear antigen antibodies, and anti-cyclic citrullinated peptide antibodies were negative. A computed tomography (CT) scan of the chest was normal. Although the patient did not have abdominal symptoms or signs, a colonoscopy was performed that showed dispersed irregular ulcers in the cecum, ileocecum and ascending colon (Fig. 2A). Histopathology from the ascending colon ulcer showed mucosal medium with lymphocytes, plasma cells, neutrophil infiltration, and erosion exudate (Fig. 2B). A biopsy of the intestinal tissue was negative for acid-fast staining. The clinical symptoms combined with the laboratory and diagnostic test results were consistent with a diagnosis of intestinal BD. A sputum smear test and chest radiograph were done prior to therapy in order to rule out the presence of active TB. T-spot.TB test was negative, and a bone marrow test was normal. She was treated with corticosteroids (30 mg/d) in combination with infliximab (antitumor necrosis factor-alpha) by intravenous infusion (200 mg per dose). Her symptoms improved: body temperature was normal, and the oral and genital ulcers and erythema nodosum disappeared.

**Figure 3:**
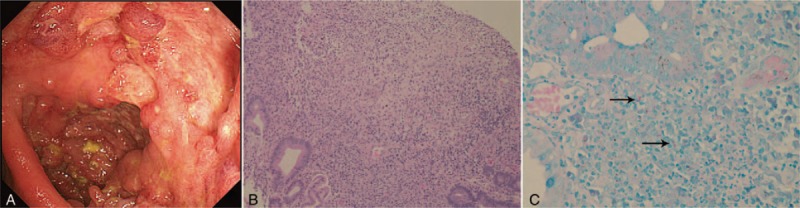
Colonoscopic finding and histopathologic examination after infliximab three months later. A, colonoscopic image of the patient showed multiple ulcers and hyperplastic polyp in ileocecus. B, histopathology from the ileocecus shows the surface having erosion exudate and necrosis, gland structure disorder, goblet cells decreased, mass lymphocytes and neutrophils infiltrate and granulomatous formation in mesenchyma. C, Positive acid fast staining in histologic finding from the second colonoscopy (black arrow).

**Figure 4:**
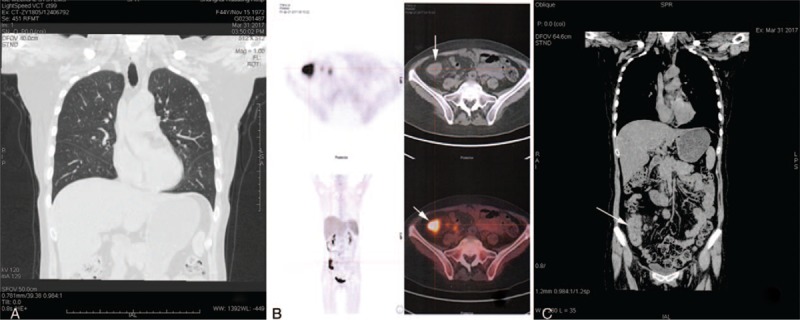
(A) Pulmonary CT examination was normal. (B) PET/CT examination. The ileocecus and ascending colon show increased local sugar metabolism (SUV = 14.1). (C) Abdominal CT examination shows a thickening of the ileocecal wall (white arrow). PET/CT = positron emission tomography/computed tomography); SUV = standard uptake value.

Following three doses of infliximab (4 months later), the patient experienced high fever for 3 days without cough and gastrointestinal discomfort, or any other symptoms of BD. Erythrocyte sedimentation rate was 55 mm/h, C-reactive protein was 42.5 mg/L, and hemoglobin was 115 g/dl. Chest radiograph was normal (Fig. 4A). A second endoscopy (4 months after the first colonoscopy) showed multiple ulcers and a hyperplastic polyp in the ileocecus (Fig. 3A). Histopathology from the ileocecus showed an erosion exudate and necrosis on the surface, a gland structure disorder, decreased goblet cells, a mass of lymphocytes, and infiltration of neutrophils and granulomatous formation in the mesenchyma (Fig. 3B). Histologic findings were positive for acid-fast staining (Fig. 3C). Positron emission tomography/computed tomography scans showed increased local sugar metabolism (standard uptake value = 14.1) in the ileocecus and ascending colon (Fig. 4B). Abdominal CT examination showed a thickening of the ileocecal wall (Fig. 4C). T-spot.TB test was positive (control well = 0, ESAT-6 = 0, CFP-10 > 30). A diagnosis of ulcerative intestinal TB was made and she was treated with isoniazid, rifapentine, ethambutol, and pyrazinamide. After 3 days, her temperature was normal. At follow-up about three months, the therapy, including anti-tuberculosis drugs and thalidomide, the therapy continued as before and her condition was stabilized.

## 3 Discussion

TNF-α is a cytokine that plays an important role in the mediation of inflammation and immune regulation. Cytokines are required for the inflammatory response against intracellular organisms, especially TB. TNF-α is involved in the pathological changes of latent tuberculous infection, especially in maintaining the formation and function of the granuloma, which prevents mycobacteria from disseminating into the blood.^[7]^ Over the last decade, a considerable amount of literature has accumulated regarding the use of infliximab for intestinal BD.^[8–10]^ Our previous data showed that infliximab is effective and safe for induction and maintenance therapy in Chinese patients with moderate-to-severe active intestinal BD.^[11]^ Suppressing the action of TNF-α can help relieve the symptoms of intestinal BD by reducing the inflammatory process; however, at the same time, it weakens the immune response to microbes such as tubercle bacilli. This could be the reason that patients develop TB following infliximab therapy.

A variety of adverse reactions have been observed with infliximab therapy. One of them is that it has been recognized as a risk factor for active tuberculosis in patients with autoimmune diseases, though the risk has been reported with widely varying rates of incidence.^[12]^ Some reported the incidence to be as low as 0.09%, while other studies (such as Malaviya et al) have reported an incidence as high as 9.4%.^[13,14]^ One study of 70 cases of TB following infliximab therapy identified 30 as pulmonary TB and 40 as extrapulmonary disease. Active tuberculosis may develop soon after the initiation of treatment with infliximab, within a median period of 12 weeks, and is likely due to reactivation of latent TB.^[15]^ We report a case of active TB after two months (8 weeks) infliximab treatment.

Intestinal BD and ulcerative intestinal TB are totally 2 different kinds of diseases but shared similar clinical manifestions. The most frequently involved location is the ileocecal area. Typical ulcerations of intestinal BD are described as a single or few, large, discrete, and round or oval shaped ulcerations in the ileocecal area.^[16]^ But it's hard to distinguish if it is atypical. They all may cause serious complications, such as perforation, and decreased quality of life, the management of these two different diseases are opposite. Tuberculosis should be excluded before the diagnosis of intestinal BD.

Intestinal TB generally is thought to be rare, and accounts for 2% of all tuberculosis cases worldwide.^[17]^ Approximately 50% of the cases of TB following infliximab treatment were extrapulmonary; intestinal TB is even rarer. Karagiannis et al reported a case of intestinal TB in an patient with rheumatoid arthritis who was on infliximab treatment.^[18]^ Intestinal TB presents a diagnostic challenge, given its non-specific clinical presentation and tendency to mimic other abdominal pathologies, such as inflammatory bowel disease and malignancy.^[19,20]^

## 4 Conclusions

The uniqueness of our case lies in the following points. First, she had no history of TB, and no evidence of it per her chest CT scan. Second, an interferon-γ releasing assay by means of the T-spot.TB test showed negative results at the early onset of the disease. Third, histopathologic examination showed no caseous necrosis granuloma. The fourth, also the most interesting finding, is that the patient was without gastrointestinal symptoms from the beginning of the disease onset. Our center has found that approximately 15% of patients with BD have no gastrointestinal symptoms, and yet gastrointestinal ulcer is identified by endoscopy (data have not been published). These unusual clinical symptoms can result in misdiagnosis or delayed diagnosis. All patients should be screened for tuberculosis exposure prior to starting treatment with infliximab. This case demonstrates the importance of close monitoring patients on infliximab for subsequent TB, even if initial screening tests have been negative.

## Author contributions

**Funding acquisition:** Jianlong Guan.

**Investigation:** Yan-li Yang.

**Validation:** Yan Shen, Jianlong Guan.

**Writing – original draft:** Yan Shen.

**Writing – review & editing:** Hai-fen Ma, Yan-li Yang.
